# Inhibitory activity of hinokitiol against biofilm formation in fluconazole-resistant *Candida* species

**DOI:** 10.1371/journal.pone.0171244

**Published:** 2017-02-02

**Authors:** Dae Jin Kim, Min Woo Lee, Jeong Su Choi, Seung Gwan Lee, Jee Yoon Park, Suhng Wook Kim

**Affiliations:** 1 Department of Integrated Biomedical and Life Sciences, Graduate School, Korea University, Seoul, Korea; 2 Department of Obstetrics and Gynecology, Kangbuk Samsung Hospital, Sungkyunkwan University School of Medicine, Seoul, Korea; Massachusetts General Hospital, UNITED STATES

## Abstract

The aim of this study was to investigate the ability of hinokitiol to inhibit the formation of *Candida* biofilms. Biofilm inhibition was evaluated by quantification of the biofilm metabolic activity with XTT assay. Hinokitiol efficiently prevented biofilm formation in both fluconazole-susceptible and fluconazole-resistant strains of *Candida* species. We determined the expression levels of specific genes previously implicated in biofilm development of *C*. *albicans* cells by real-time RT-PCR. The expression levels of genes associated with adhesion process, *HWP1* and *ALS3*, were downregulated by hinokitiol. Transcript levels of *UME6* and *HGC1*, responsible for long-term hyphal maintenance, were also decreased by hinokitiol. The expression level of *CYR1*, which encodes the component of signaling pathway of hyphal formation-cAMP-PKA was suppressed by hinokitiol. Its upstream general regulator *RAS1* was also suppressed by hinokitiol. These results indicate that hinokitiol may have therapeutic potential in the treatment and prevention of biofilm-associated *Candida* infections.

## Introduction

*Candida* species are opportunistic pathogens that live commensally within the human body. The prevalence of *Candida* infections, which ranges from superficial to deep-seated invasive candidiasis, is increasing at an alarming rate especially among immunocompromised individuals [1]. The most common isolated *Candida* species in clinical fungal invasive infection is *Candida albicans*, followed by *C*. *tropicalis*, *C*. *parapsilosis* and *C*. *glabrata* [2,3]. While *C*. *albicans* remains the most frequently isolated species, its incidence is declining and the frequency of other species is increasing. *C*. *tropicalis* mostly affects neutropenic patients and individuals with haematological malignancies [4]. *C*. *glabrata* affects mostly older patients, diabetics or individuals pre-exposed to azoles or echinocandins, and is rarely isolated from neonates and young children [5]. *Candida* species are heterogeneous, so understanding their phylogenetic differences may help to explain the reasons underlying variations of prevalence in *Candida* species. The most closely related species are *C*. *albicans* and *C*. *tropicalis* whereas *C*. *glabrata* is more closely related to *Saccharomyces cerevisiae* [6]. Most manifestations of candidiasis are associated with biofilm formation occurring on the surfaces of host tissues and medical devices [7,8]. Moreover, the most important feature of *Candida* biofilms is its role in increasing tolerance to conventional antifungal therapy. Forming biofilms is the answer of microorganisms to hostile environments. The optimal protection of the embedded cells against noxious agents, e.g., antibiotics and the immune system, is the main reason why biofilm infections are so difficult to treat. It has been found that for the eradication of pathogens from biofilms more than 1000 times higher antibiotic concentrations were required than for the same strain living in planktonic form in the serum [9]. The most common agent used in clinic against candidiasis are fluconazole and the candins. However, poor activity of fluconazole against *Candida* biofilms is a well-known phenomenon, which may decrease efficacy of fluconazole treatment [10]. Also, prolonged exposures to fungistatic fluconazole can result in the emergence of acquired resistant isolates. The spread of drug-resistant pathogens is one of the most serious threats to successful treatment of microbial diseases. Therefore, it has become essential to develop novel and potent antifungal for the treatment of fluconazole-resistant *Candida* infections.

In recent decades plant essential oils and their components have attracted increased interest and consequently have been extensively investigated. Essential oils are aromatic oily liquids obtained from plant materials. The strong antibacterial and antifungal activities of essential oils and their major components have been described [11–13]. Hinokitiol, also known as β-thujaplicin, is a bioactive compound of aromatic seven-member tropolone and a component of essential oils first isolated from the heart wood of *Chamacyparis taiwanensis*. Hinokitiol has a wide range of biochemical and pharmacological activities, and antibacterial [14], antifungal [15, 16], antiviral [17], antitumor [18], and insecticidal [19] activities. Here we demonstrate the antifungal and antibiofilm activities of hinokitiol on fluconazole-susceptible and fluconazole-resistant strains of *Candida* species. We also investigated the inhibitory effect of hinokitiol on the signals for *C*. *albicans* biofilm formation using real-time RT-PCR.

## Materials and Methods

### Microorganisms, reagents and growth medium

*C*. *albicans* (ATCC 90028, 90029), *C*. *glabrata* (ATCC 90030) and *C*. *parapsilosis* (ATCC 90018) were purchased from the American Type Culture Collection. *C*. *guilliermondii* (KCMF 20104), *C*. *parapsilosis* (KCMF 20154), fluconazole-resistant *C*. *albicans* (KCMF 20017), fluconazole-resistant *C*. *glabrata* (KCMF 20161) and fluconazole-resistant *C*. *tropicalis* (KCMF 20197) were obtained from the Korean Collection of Medical Fungi. For susceptibility to fluconazole, *C*. *albicans*, *C*. *guilliermondii*, *C*. *parapsilosis*, *C*. *tropicalis* with MIC ≥8 μg/ml were considered to be resistant and *C*. *glabrata* with MIC ≥64 μg/ml were considered to be resistant. To prepare suspensions of budding cells, all of the strains were propagated in yeast extract peptone dextrose (YPD) medium and incubated overnight in an orbital shaker at 30°C. Stock solutions of hinokitiol, thymol, geraniol, citral, carvacrol, menthol, eugenol, linalool, camphor and fluconazole (Sigma-Aldrich, St Louis, MO, USA) were prepared utilizing dimethyl sulfoxide (DMSO) as the solvent, and stored at − 80°C until use. The final concentration of DMSO in the test solutions was less than 0.5%. Commercially available human bone marrow-derived mesenchymal stem cells (MSCs) were purchased from Lonza (Walkersville, MD, USA).

### Determination of minimum inhibitory concentration (MIC) and minimum fungicidal concentration (MFC)

MIC for planktonic cells was determined according to Clinical and Laboratory Standards Institute M27-A3 document with slight modifications [20]. In brief, serial two-fold dilutions of each compound were prepared and added to 96-well flat-bottomed microtiter plates. The test strains were suspended in RPMI 1640 medium at a final density of 0.5–2.5 × 10^3^ CFU/ml and added to the test wells containing diluted test compounds. All the plates were incubated at 30°C for 48 h and the absorbance at 620 nm was measured using a SpectraMax 190 microplate reader (Molecular Devices, Downingtown, PA, USA) to assess cell growth. The lowest concentration of the test compound which cause ≥50% reduction in the absorbance compared to that of control was considered the MIC. To determine MFC, 50 μl of the cell suspension was taken from each clear well containing test compounds at a concentration above the MIC and spread on YPD agar. These plates were incubated for 48 h at 30°C and observed for the presence of colonies. MFC was determined to be the lowest concentration that did not allow visible growth on the surface of the agar.

### Biofilm formation and treatment

*Candida* biofilms were developed on the surface of 96-well polystyrene plates as described previously [21]. In brief, cell suspensions of 1 × 10^5^ cells/ml in RPMI 1640 medium were prepared and 200 μl were added to select wells and incubated at 37°C for 1 h. Following this initial 1-h adhesion period, the non-adherent cells were removed by washing with PBS, after which fresh RPMI 1640 medium containing various hinokitiol concentrations was added to the adherent cells, and the plates were incubated at 37°C for 24 h. The effects of hinokitiol on *Candida* biofilm formation were estimated using a semi-quantitative 2,3-bis-(2-methoxy-4-nitro-5-sulfophenyl)-2H-tetrazolium-5-carboxanilide (XTT) reduction assay, as described below. To analyze the effects of hinokitiol on pre-formed biofilms, *Candida* biofilms were established for 24 h at 37°C on the surfaces of microtiter plates using the protocol described above. After washing with PBS, fresh RPMI 1640 medium containing various hinokitiol concentrations was added to the selected wells and incubated at 37°C for an additional 24 h. The effects of hinokitiol on the pre-formed biofilms were estimated using the XTT reduction assay, as described below.

### Biofilm quantitation by XTT assay

Biofilm growth was quantified using a XTT metabolic assay [12]. Briefly, XTT (Sigma-Aldrich) was prepared as a saturated solution in PBS and stored at − 70°C. Prior to use, menadione solution was prepared in acetone and added to the XTT to a final concentration of 1 μM. A 100 μl aliquot of the XTT-menadione solution was then added to each pre-washed biofilm, and to control wells to measure background XTT levels. The plates were incubated in the dark for 1 h at 37°C, and then the absorbance was measured at 490 nm using a microtiter plate reader. The XTT values of the growth control were normalized to 100%, and the results were expressed as percentages of the control absorbance values. The concentration of hinokitiol producing ≥50% lowering in relative metabolic activity was considered the MIC for biofilm formation.

### Scanning electron microscopy (SEM)

*C*. *albicans* biofilms were formed on custom-made plastic coverslips. To observe the inhibitory effect of hinokitiol on biofilm formation, *C*. *albicans* suspensions were added to plastic coverslips in 6-well culture plates and incubated at 37°C for 1 h. Following the initial 1-h adhesion step, hinokitiol was added, and the plates incubated for 24 h. After biofilm formation and hinokitiol treatment, the biofilms were washed with PBS and fixed overnight in 2% glutaraldehyde in PBS. The coverslips were washed twice with PBS and then placed in 1% osmium tetroxide for 1 h. Samples were subsequently washed with distilled water, dehydrated in a series of ethanol solutions (70% for 10 min, 95% for 10 min and 100% for 20 min), and air-dried overnight in a desiccator prior to sputter coating with palladium. The surface topographies of the *C*. *albicans* biofilms were viewed with a scanning electron microscope (JEOL Ltd., Tokyo, Japan).

### Cell cytotoxicity test

Cytotoxicity of test compounds was determined in human MSCs. A viability assay based on the conversion of 3-(4,5-dimethylthiazol-2-yl)-2,5-diphenyltetrazolium bromide (MTT) was performed to test cytotoxicity and cell viability based on the conversion of yellow tetrazolium salt to an insoluble purple formazan product. MIC of test compounds were prepared in 96 well flat-bottom tissue culture plates using Dulbecco’s modified Eagle’s medium containing 10% fetal bovine serum. Each well was inoculated with 2×10^4^ cells. After two days of incubation at 37°C, MTT solution was added and cells were incubated for 2 h. After the supernatant was eliminated and DMSO was added to each well to dissolve the formazan, the absorbance at 570 nm was measured with a microtiter plate reader.

### Quantitative real time RT-PCR analysis of *C*. *albicans* specific genes

Biofilms of *C*. *albicans* (ATCC 90028) were grown in the absence or presence of hinokitiol (1/2 × MIC) in 6-well plates as described above. After washing with PBS, biofilm cells were removed from the bottom of the plates with a sterile scraper, and the total RNA was purified using an RNeasy kit (Qiagen, Hilden, Germany), in accordance with the manufacturer's instructions. One microgram of template was reverse-transcribed with Super Script First Strand (Invitrogen, Life Technologies, Carlsbad, CA, USA). Expression of hyphal-specific and biofilm-associated genes (*HWP1*, *ALS3*, *UME6* and *HGC1*) and their transcriptional regulators (*RAS1*, *CYR1* and *EFG1*) was analyzed. The relative expression levels of the target genes were analyzed using a Rotor-Gene Q system (Qiagen). Platinum SYBR Green PCR Master Mix (Invitrogen) was used to monitor the amplified product in real time, following the manufacturer's protocol. Primers for the tested genes were taken from the literature (Table 1). PCR consisted of denaturation at 95°C for 10 min, followed by 40 cycles of amplification (95°C for 10 s, 55°C for 10 s, and 72°C for 10 s) and quantification. The expression of 18S rRNA was used for normalization and to calculate the relative changes in target gene expression. Gene expression is given in relative values, setting the expression level of the untreated control to 1 for each gene. The assays were performed in triplicate and repeated three times.

**Table 1:** Primer sequences used for RT-PCR experiments.

Primer name	Primer sequence (5′–3′)	Reference
18S rRNA-F	CACGACGGAGTTTCACAAGA	[22]
18S rRNA-R	CGATGGAAGTTTGAGGCAAT	[22]
RAS1-F	CCCAACTATTGAGGATTCTTATCGTAAA	[23]
RAS1-R	TCTCATGGCCAGATATTCTTCTTG	[23]
CYR1-F	CCAACAAACGACCAAAAGGT	[24]
CYR1-R	TCTTGAACTGCCAGACGATG	[24]
EFG1-F	GCCTCGAGCACTTCCACTGT	[25]
EFG1-R	TTTTTTCATCTTCCCACATGGTAGT	[25]
ALS3-F	CAACTTGGGTTATTGAAACAAAAACA	[25]
ALS3-R	AGAAACAGAAACCCAAGAACAACC	[25]
HWP1-F	GCTCCTGCTCCTGAAATGAC	[26]
HWP1-R	CTGGAGCAATTGGTGAGGTT	[26]
UME6-F	ACCACCACTACCACCACCAC	[27]
UME6-R	TATCCCCATTTCCAAGTCCA	[27]
HGC1-F	GCTTCCTGCACCTCATCAAT	[24]
HGC1-R	AGCACGAGAACCAGCGATAC	[24]

### Statistical analysis

Each experiment was performed at least in triplicate. The statistical analysis was performed using one-way ANOVA test. A P-value < 0.05 was considered to be statistically significant.

## Results

### Fungal susceptibility assay

We first tested antifungal activity of hinokitiol against *C*. *albicans* (ATCC 90028) using the MIC and MFC in broth microdilutions. Eight terpenoids known to have strong antifungal activities against *C*. *albicans* were also selected and their MIC and MFC were compared with those of hinokitiol [28]. The antifungal agent fluconazole was used as a positive control. As shown in Table 2, hinokitiol was the most active substance in inhibiting planktonic growth of *C*. *albicans* (ATCC 90028) with a MIC value of 1.6 μg/ml. Fluconazole showed a slightly lesser MIC (2.5 μg/ml) than hinokitiol. The eight terpenoids showed 120–2000-times lower inhibitory activity than that of hinokitiol against *C*. *albicans* (ATCC 90028). In a decreasing scale of activity, they were thymol, geraniol, citral, carvacrol, menthol, eugenol, linalool and camphor. Cells from the microdilution assays after incubation with hinokitiol and terpenoids at various concentrations were plated on agar to determine the colony forming units for MFC determination. Although the MFC of hinokitiol was higher than the MIC, hinokitiol was the most potent in killing *C*. *albicans* cells, with a MFC value of 100 μg/ml.

**Table 2:** Antifungal activity of hinokitiol, fluconazole and selected terpenoids against planktonic growth of *C*. *albicans* (ATCC 90028).

Test compound	MIC (μg/ml)	MFC(μg/ml)
Hinokitiol	1.6	100
Thymol	193	1544
Geraniol	352	2813
Citral	355	1421
Carvacrol	390	1562
Menthol	800	3200
Eugenol	854	1707
Linalool	2784	>2784
Camphor	≥3200	>3200
Fluconazole	2.5	>320

We next tested antifungal effects of hinokitiol in both fluconazole-susceptible and fluconazole-resistant strains of *Candida*. MIC values of hinokitiol and fluconazole against the nine strains are shown in Table 3. MIC of hinokitiol ranged from 0.78 μg/ml to 6.3 μg/ml; the growth of all strains was inhibited. More importantly, hinokitiol demonstrated a strong anti-candidal effect against fluconazole-resistant strains. Hinokitiol showed potent antifungal activity toward the fluconazole-resistant strains, *C*. *albicans* (KCMF 20017), *C*. *glabrata* (KCMF 20161) and *C*. *tropicalis* (KCMF 20197) with a MIC value of 1.6 μg/ml, 0.78 μg/ml and 3.1 μg/ml, respectively. Hinokitiol also showed fungicidal activity against planktonic cells of all assayed *Candida* species including fluconazole-resistant strains, with MFC values between 100 and 200 μg/ml. These data implicated hinokitiol as an effective antimicrobial agent against both fluconazole-susceptible and fluconazole-resistant strains of *Candida* species.

**Table 3:** Antifungal activity of hinokitiol against *Candida* species and the effects of hinokitiol on *Candida* biofilm formation and on the mature biofilms.

	Antifungal activity				Effects of hinokitiol on biofilm	
	Hinokitiol (μg/ml)		Fluconazole (μg/ml))		Biofilm formation (μg/ml))	Mature biofilms (μg/ml))
Test strains	MIC	MFC	MIC	MFC	MIC	MIC
*C*. *albicans* ATCC 90028	1.6	100	2.5	>320	3.1	400
*C*. *albicans* ATCC 90029	1.6	100	2.5	>320	3.1	400
*C*. *albicans* KCMF 20017	1.6	100	160	>320	3.1	200
*C*. *glabrata* ATCC 90030	0.78	100	2.5	>320	3.1	50
*C*. *glabrata* KCMF 20161	0.78	100	80	>320	3.1	50
*C*. *guiliermondi* KCMF 20104	6.3	200	2.5	>320	6.3	12.5
*C*. *parapsilosis* ATCC 90018	6.3	200	2.5	>320	12.5	25
*C*. *parapsilosis* KCMF 20154	6.3	200	2.5	>320	6.3	25
*C*. *tropicalis* KCMF 20197	3.1	200	160	>320	3.1	400

### Effects of hinokitiol on biofilm formation and on the maintenance of pre-formed biofilms

To assess the biofilm inhibitory activity of hinokitiol, a microtiter-based colorimetric assay was used for both fluconazole-susceptible and fluconazole-resistant strains of *Candida* species grown as biofilms against hinokitiol. MICs for biofilm formation were determined at 50% inhibition in relative metabolic activity using the XTT reduction assay. Consistent with its antifungal activity against planktonic cells, hinokitiol exhibited an inhibitory effect on biofilm formation of all fluconazole-susceptible and fluconazole-resistant strains of *Candida* species, with MIC values between 3.1 and 12.5 μg/ml (Table 3). Hinokitiol also showed inhibitory activity against mature biofilms with MIC values between 12.5 and 400 μg/ml (Table 3), consistent with the prior observation that mature biofilms are more resistant to antifungal drugs [21].

**Fig 1:**
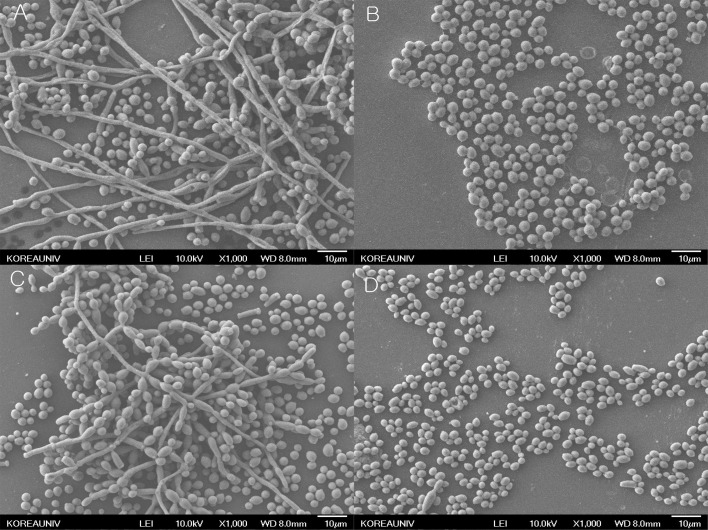
Representative SEM images showing the topography of both fluconazole-susceptible and fluconazole-resistant *C*. *albicans* biofilms. Fungal biofilms were formed on plastic coverslips, processed, and coated with palladium before viewing. The magnification level is 500 ×. A) Biofilm formation of fluconazole-susceptible *C*. *albicans* (ATCC 90028) in the absence of hinokitiol. B) Biofilm formation of fluconazole-susceptible *C*. *albicans* (ATCC 90028) in the presence of 3.1 μg/ml of hinokitiol. C) Biofilm formation of fluconazole-resistant *C*. *albicans* (KCMF 20017) in the absence of hinokitiol. D) Biofilm formation of fluconazole-resistant *C*. *albicans* (KCMF 20017) in the presence of 3.1 μg/ml of hinokitiol.

The effects of hinokitiol on both fluconazole-susceptible and fluconazole-resistant *C*. *albicans* biofilms were visually confirmed by SEM. For non-treated control cells (Fig 1A and 1C), both fluconazole-susceptible and fluconazole-resistant *C*. *albicans* biofilms formed on the plastic coverslips consisted of multilayer filamentous cells and scattered colonies. Compared to the non-treated control, no filamentous cells were observed in both fluconazole-susceptible and fluconazole-resistant *C*. *albicans* cultures treated with MIC of hinokitiol for 24 h after the 1-h adhesion step, and scattered colonies, mainly composed of yeast cells, were visualized by SEM (Fig 1B and 1D). These observations are consistent with the inhibition of biofilm formation as determined by XTT reduction assays. Thus SEM analysis supported the biofilm quantification results.

### Cell cytotoxicity test

**Fig 2:**
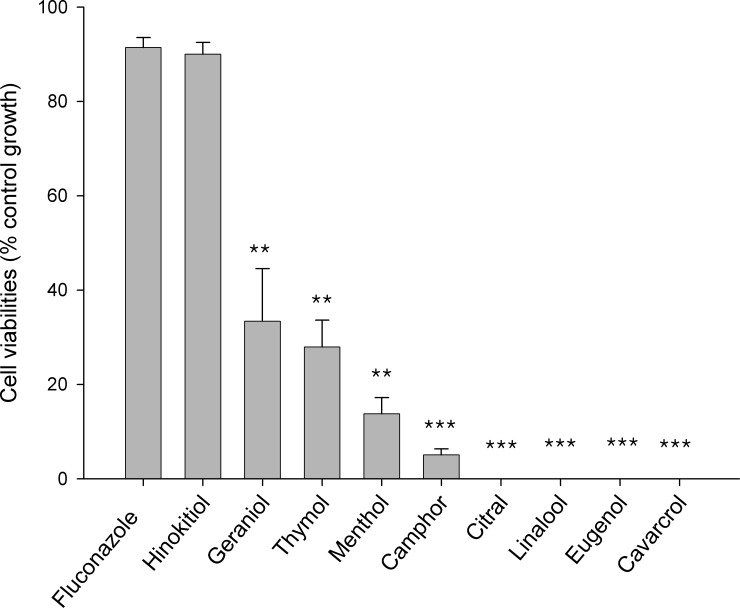
Effects of hinokitiol, fluconazole and selected terpenoids on the proliferation of MSCs. MSCs were treated with MIC of hinokitiol, fluconazole and select terpenoids for 48 h. Cell proliferation was determined by MTT assay. Data are the means ± SD of triplicate determinations. **P<0.01 and ***P<0.001.

The cytotoxicity of hinokitiol, eight terpenoids and fluconazole on human bone marrow-derived MSC was investigated using the MTT assay. MSC survival was reduced by >67% upon treatment with MIC concentrations of the eight terpenoids, showing that MICs of all the terpenoids tested in this study for *C*. *albicans* growth were toxic to MSC (Fig 2). However, only 9% and 10% of the MSC populations was killed by treating with MIC concentrations of fluconazole and hinokitiol, respectively, indicating that fluconazole and hinokitiol have similar low cytotoxic activity (Fig 2).

### Effect of hinokitiol on the expression of hyphae-associated genes in *C*. *albicans*

**Fig 3:**
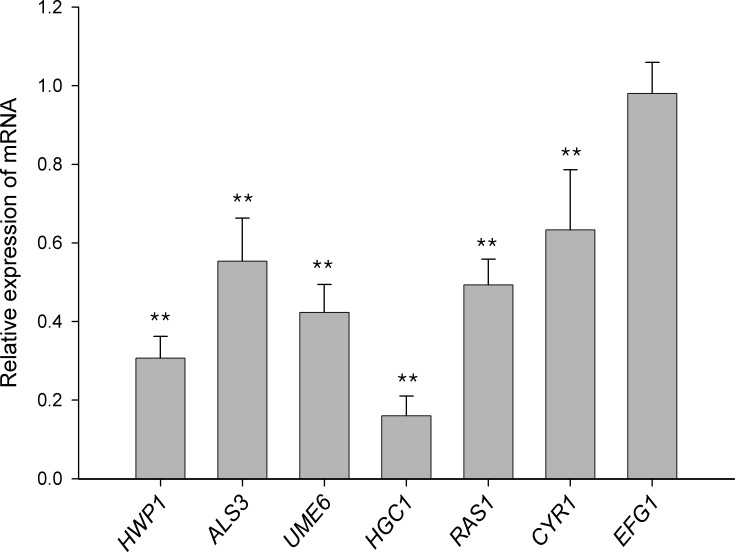
Quantitative real time RT-PCR analysis of *C*. *albicans* specific genes. *C*. *albicans* biofilms were formed in the presence or absence of hinokitiol (1/2 × MIC) and expression of the target genes was determined by quantitative real-time RT-PCR. Housekeeping gene 18S rRNA was used for normalization. The expression level of the untreated sample was set to 1 for each gene. Data are the means ± SD of triplicate determinations. **P<0.01.

To elucidate the potential molecular mechanism behind the ability of hinokitiol to prevent growth of *C*. *albicans* biofilms, we analyzed the changes in the gene expression levels of *C*. *albicans* cells in biofilms exposed to hinokitiol (Fig 3). The expression levels of specific genes previously implicated in biofilm development of *C*. *albicans* cells were determined by real-time RT-PCR, including genes in the adhesion process (*HWP1* and *ALS3*), and hyphae formation and maintenance (*UME6* and *HGC1*). Expression of hyphal transcriptional regulators in the cAMP-dependent protein kinase pathway (*RAS1*, *CYR1* and *EFG1*) were also assayed. The transcription levels of hyphae-specific genes that encode products that function as surface adhesins, such as *HWP1* (hyphal cell wall protein) and *ALS3* (agglutinin-like sequence), were suppressed by hinokitiol treatment during the early stage of biofilm development (Fig 3). In addition, the expression levels of the *UME6* and *HGC1* genes, which are associated with long-term hyphal maintenance, and *CYR1* and *RAS1* were suppressed by hinokitiol. *HWP1* and *ALS3* are downstream components of the cAMP-PKA pathway and are positively regulated by *EFG1*. Although the expression level of *EFG1* was not affected by hinokitiol treatment in our study, the upstream components, *CYR1* and *RAS1*, were downregulated when the cells were treated with hinokitiol (Fig 3). This is consistent with the findings that hinokitiol-mediated inhibition of *C*. *albicans* growth may occur through the inhibition of the *RAS1* signal pathway [16]. These results indicate that hinokitiol treatment may affect biofilm formation by reducing the levels of adhesins, interfering with hyphae formation due to the disruption of the cAMP-PKA pathway and restricting the long-term maintenance of hyphae.

## Discussion

Cells in biofilms are much better protected against noxious agents than free-living cells. From a medical viewpoint, the most critical feature of biofilm growth is the development of antimicrobial resistance by the microorganisms that constitute the biofilm [29]. The elimination of mature biofilms presents a therapeutic challenge in the management of device-associated *Candida* infections [30]. In this study, hinokitiol effectively inhibited the generation of *Candida* biofilms. At higher concentrations, hinokitiol also showed inhibitory activity against mature biofilms. Hinokitiol exhibited inhibitory activity against mature biofilms of *C*. *guiliermondi* and *C*. *parapsilosis* with MIC values of 12.5 and 25 μg/ml, respectively (Table 3). These MIC values were 2 to 4 times higher for mature biofilms than for planktonic cells. However, hinokitiol exhibited inhibitory activity against mature biofilms of *C*. *albicans*, fluconazole-resistant *C*. *albicans*, *C*.*glabrata* and *C*. *tropicalis* with MIC values of 400, 200, 50 and 200 μg/ml, respectively (Table 3). These MIC values were 64 to 250 times higher for mature biofilms than for planktonic cells. Lower activities are observed for other terpenoids, such as thymol, carvacrol and eugenol. These terpenoids showed 2.5-times lower inhibitory activity than that of hinokitiol against *C*. *albicans* biofilms [28, 31]. Echinocandins have shown some effectiveness against *in vivo* mature *C*. *albicans* biofilms [32]. Echinocandins were not utilized and this is a limitation of our study. Also, further evaluation is required to determine the antibiofilm activity of hinokitiol *in vivo*.

In conclusion, we demonstrate that hinokitiol exhibits excellent antifungal activity against all fluconazole-susceptible and fluconazole-resistant strains of *Candida* species tested. We also demonstrate that hinokitiol prevents biofilm formation and reduces both fluconazole-susceptible and fluconazole-resistant *Candida* biofilms that have been allowed to develop for 24 h. In addition, hinokitiol showed low cytotoxic activity against human bone marrow-derived MSCs. These results suggest that hinokitiol could provide an improved and safe clinical approach in treating biofilm-associated *Candida* infections.
